# Assessment of the Prevalence of Anxiety and Depressive Symptoms, Life Satisfaction and Quality of Life Among Women in the Maternity Ward with the Impact of the COVID-19 Pandemic

**DOI:** 10.3390/jcm14176279

**Published:** 2025-09-05

**Authors:** Joanna Furman, Beata Łabuz-Roszak, Ewa Niewiadomska

**Affiliations:** 1 Department of Environmental Health Risk Factors, Faculty of Public Health in Bytom, Medical University of Silesia in Katowice, 41-902 Bytom, Poland; jfurman@sum.edu.pl; 2 Department of Neurology, St. Jadwiga Regional Specialized Hospital, Institute of Medical Sciences, University of Opole, 45-221 Opole, Poland; 3 Department of Biostatistics, Faculty of Public Health in Bytom, Medical University of Silesia in Katowice, 41-902 Bytom, Poland; eniewiadomska@sum.edu.pl

**Keywords:** anxiety, COVID-19 pandemic, depression, life satisfaction, postpartum depression, quality of life

## Abstract

**Background:** The postpartum period may predispose to a higher prevalence of mental health disorders. The aim of the study was to assess the prevalence of anxiety and depressive symptoms, life satisfaction, and quality of life in breastfeeding women in the maternity ward in relation to specific medical and social factors. **Methods:** The study group consisted of 304 female patients from the maternity ward of the Multispecialist District Hospital in Tarnowskie Góry, Poland. The research tool included four questionnaires: Hospital Anxiety Depression Scale, Edinburgh Postnatal Depression Scale, Satisfaction with Life Scale, and Euro-Quality of Life Questionnaire. **Results:** The majority of women in the maternity ward reported good psychological well-being. Anxiety symptoms affected 11.9% of postpartum women, depressive symptoms—7.3%, and symptoms of postpartum depression—5.9%. The COVID-19 pandemic caused an increase in anxiety and depressive disorders (relative differences-expressed as a percentage). Women who gave birth by cesarean section were more likely to declare problems that negatively impacted their quality of life and health than those who gave birth naturally (OR = 1.28, 95% CI: 0.77–2.11). The risk of experiencing anxiety and depressive symptoms, as well as postpartum depression symptoms decreased as self-rated health increased (OR = 0.96, 95% CI: 0.94–0.99; OR = 0.96, 95% CI: 0.94–0.99; OR = 0.96, 95% CI: 0.93–0.98, respectively). Higher level of life satisfaction was associated with higher levels of education and economic status, attendance at childbirth classes, and a higher self-assessment of health (OR = 4.1, 95% CI: 1.6–10.51; OR = 2.96, 95% CI: 1.41–6.24; OR = 1.99, 95% CI: 1.13–3.49; OR = 1.01, 95% CI: 1.01–1.04, respectively). **Conclusions:** Screening for mental disorders during the postpartum period enables the early identification of symptoms and the implementation of appropriate treatment. Women who give birth by cesarean section and have medical complaints should be given special follow-up care. Health policy should ensure wider access to psychological and psychiatric care during the postpartum period.

## 1. Introduction

The postpartum period is a time of many changes in a woman’s life related to caring for her newborn and adjusting to a new role. The Social Readjustment Rating Scale (SRRS), developed by Thomas Holmes et al. [1], is used to measure stress experienced in life. It classifies life events on a 100-point scale. These include pregnancy and childbirth. These life events were ranked at 40 and 39 points, respectively [1]. Factors such as fatigue, restricted social contact or interruption in professional activity can reduce quality of life, resulting in temporary and reversible emotional fluctuations, but can also develop into mental disorders and even lead to mental illness. The most common mental disorders of the postpartum period include postpartum blues, postpartum depression, postpartum psychosis, post-traumatic stress disorder, mother-child relationship disorders or anxiety disorders [2,3].

Postpartum blues (baby blues), also known as postpartum sadness, is the most common, but also the least severe, mental disorder of the postpartum period. It is estimated that the percentage of women experiencing postpartum blues may be between 50% and 85%. It is the body’s physiological response to the stress of pregnancy and childbirth, as well as the hormonal changes that occur in the postpartum period. However, increased pospartum sadness is a risk factor for postpartum depression. Postpartum blues can begin as early as the first day after delivery, with an intensification of symptoms usually oberved around days 5–7, when the greatest hormonal levels changes occur (a decrease in progesterone, estrogen, and cortisol, and an increase in prolactin). It typically lasts for about two weeks; in special cases, it can last up to three months. Symptoms include mainly anxiety, irritability, a moderate lowering of mood, tearfulness, excessive sensitivity to stimuli, a decrease in appetite, sleep disturbances, and headaches. Sometimes there are feelings of hostility toward the partner [3,4,5,6].

Postpartum depression (PPD) is defined as depression that occurs in the postpartum period. Statistically, it occurs in approximately 13–19% of women worldwide [7], around 10% of the population in high-income countries, and around 20% of women living in low- and middle-income countries [8]. There are no official epidemiological data on the prevalence of mental disorders, including PPD, in Poland [9]. The two main classification systems, the Diagnostic and Statistical Manual of Mental Disorders (DSM-5) and the International Statistical Classification of Diseases and Related Health Problems (ICD-11, formerly ICD-10), do not distinguish postpartum depression as a separate disease entity [10]. The DSM-5 classification system does not differentiate between depression during pregnancy and postpartum depression, but refers to both disorders as depressive disorder with peripartum onset [11]. In the ICD-10 classification, postpartum depression is coded F53.0—*Mild mental and behavioral disorders associated with the postpartum period, not listed elsewhere: postnatal depression and postpartum depression*. These disorders have their onset from the 6th week after delivery [12]. The updated ICD-11 classification was officially implemented on 11 February 2022. Unlike the previous classification, it includes an additional subchapter entitled “Mental or behavioral disorders associated with pregnancy, childbirth, and the puerperium”. These disorders are subdivided into those without psychotic symptoms (6E20), with psychotic symptoms (6E21), and non-specific disorders (6E2Z) [13,14]. Most experts in psychiatry define postpartum depression as a disorder that can occur at any time within the 1st year after delivery [15]. The risk of postpartum depression is influenced by psychiatric, pregnancy-related and psychosocial factors. Psychiatric factors include a previous history of mood disorders (not just postpartum depression) and a family history of depression. Pregnancy-related risk factors include an unwanted or unplanned pregnancy, previous miscarriages or stillbirths, high-risk pregnancy and severe or traumatic childbirth. Psychosocial factors may include any life changes during pregnancy and after childbirth (including positive ones) that force a woman to adapt to new conditions, the prospect of single motherhood, a low social or economic status, limited support from a partner or family and an inappropriate relationship with a mother [3,6,16,17]. The diagnostic criteria are similar to those for depression and mainly include mood swings and low mood, anxiety disorders, suicidal thoughts, circadian rhythm disturbances, avoidance of social contact, feeling of guilt, difficulty concentrating, feelings of low self-worth. Depression may be expressed and inadequate expressing sadness and tearfulness inappropriate to the situation. Additionally, a lack of interest in the child or, conversely, excessive concern for the infant, difficulty caring for the infant, fear for the child’s health, and a belief in a lack of fulfillment in the role of a mother are observed [4,5,16,18,19].

Postpartum psychosis is the most severe among postpartum disorders, but also the least common, affecting only 0.1–0.2% of postpartum women [20]. Symptoms usually appear within the first few days after delivery, occasionally in the second week and rarely later. They increase rapidly and include: insomnia or even lack of sleep for the next few days, a lack of appetite, agitation, irritability, a lack of interest in the child, disorientation, delusions and hallucinations. In extreme cases, there is a risk of suicide or infanticide. Due to its rapid progression and the risk to the health and lives of the mothers and children, postpartum psychosis often requires hospitalization [4,6]. It is more common after the birth of the first child, among single mothers, after cesarean section, and in women with a prior diagnosis of bipolar disorder or schizophrenia. Another risk factor appears to be a close relationship with someone who suffers from psychosis [4,6,21].

The Minister of Health’s Regulation of 16 August 2018 on the organizational standard of perinatal care in Poland stipulates that the assessment of the risk and severity of depressive symptoms should be performed three times: at 11–14 weeks of pregnancy (first trimester), at 33–37 weeks of pregnancy (third trimester) and during the postpartum period in the maternity ward, or during a midwife’s visit to the mother and child’s place of residence or stay [22,23]. However, the standard does not specify the type of tool to be used for screening for postpartum depression risk. The President of the Agency for Health Technology Assessment and Tariff System which is subordinate to the Polish Ministry of Health, recommends the Edinburgh Postnatal Depression Scale questionnaire [9], which is considered a global standard for assessing mental disorders in the postpartum period [24]. The Ministry of Health Recommendations also indicate high sensitivity and specificity of this type of tool [10], and Suchowiak et al. emphasize that the Edinburgh Postnatal Depression Scale is the only instrument available in the literature that has been specifically developed for screening postnatal depression [23]. Due to the lack of clear guidelines for diagnosing postpartum depression, various scales are often used [25]. One of the most frequently used tools in the general population is the Beck Depression Inventory (BDI), which can also be used to screen for perinatal depression. The BDI is used to diagnose depression, assess the severity of depressive symptoms and monitor its course and changes during treatment [26]. Research is ongoing on the use of the Mother Generated Index (MGI), which does not require linguistic validation [27]. The Postpartum Depression Screening Scale (PDSS) is another popular screening test [28]. Therefore, it seems necessary to develop specific tools for use with selected population groups to enable early detection of potential emotional disorders and the implementation of appropriate treatment.

Compared to doctors in other countries, Polish family doctors and paediatricians do not have sufficient knowledge when it comes to diagnosing mental disorders. Therefore, the main issue is not treatment, but diagnosis. This problem is exacerbated by limited access to specialists. Although visits to specialists are reimbursed by the National Health Fund, waiting times for a psychiatric consultation can be as long as several months, depending on the region [29]. Over 70% of mothers who gave birth between 2019 and 2024 said that they had not undergone the mandatory screening for postpartum depression, and only half of these women sought help from a specialist. Furthermore, 76% of respondents did not receive support following the loss of a child [30].

The following hypotheses can be formulated based on an analysis of the literature:

- Women who experience complications during childbirth (e.g., an emergency caesarean section or preterm labor) report higher levels of anxiety and depressive symptoms than those who have a routine, uncomplicated delivery;
- Women who report a lack of support from their partner or family tend to exhibit higher levels of anxiety and depressive symptoms;
- Compared to those from higher socioeconomic backgrounds, women from lower socioeconomic backgrounds report significantly higher levels of anxiety and depressive symptoms, as well as a lower quality of life.

The aim of this study was to assess the frequency of anxiety and depressive symptoms, as well as the level of life satisfaction and quality of life in breastfeeding women staying in the maternity ward, in relation to specific medical, social and epidemiological factors.

## 2. Materials and Methods

The study was conducted using the following questionnaires: the Hospital Anxiety Depression Scale (HADS), the Edinburgh Postnatal Depression Scale (EPDS), the Satisfaction with Life Scale (SWLS), and the Euro-Quality of Life Questionnaire (EQ-5D). A total of 304 patients admitted to the maternity ward at Dr. B. Hager Multispecialist District Hospital in Tarnowskie Góry, southern Poland, took part in the study between October 2019 and July 2020. Therefore, no specific inclusion criteria were established, since all patients received the questionnaires, regardless of the type of delivery or high-risk pregnancies. Above-mentioned questionnaires were given to each woman who gave birth during the study period (*n* = 389). Responses were received from 304 women, representing a response rate of 78.15%. Permission to conduct the survey among patients was obtained from the President of the Hospital Management Board. The questionnaires were completed independently, without consulting with relatives and other people. Participation in the study was voluntary and anonymous, the respondents were informed of the purpose and course of the study. The Bioethics Committee of the Medical University of Silesia in Katowice expressed its opinion that consent was not required to conduct the study (KNW/0022/KB/202/19, 14 October 2019).

### 2.1. HADS

The Hospital Anxiety and Depression Scale by Zigmond and Snaith (pl. Szpitalna Skala Lęku i Depresji według Zigmonda i Snaitha) consists of fourteen questions. The odd-numbered questions relate to anxiety levels, and the even-numbered questions relate to depression levels. Each response (100% response rate) was given a score of 0 to 3 points. The results were summed separately for each group. Scores were interpreted according to the following scale: 0–7 points—no disorders, 8–10 points—borderline states, 11–21 points—presence of anxiety and/or depressive symptoms [31]. Cronbach’s alpha for the present sample was 0.84.

### 2.2. EPDS

The EPDS scale (pl. Edynburska Skala Depresji Poporodowej) consists of ten statements with four response options. It is used for self-assessing the risk of postpartum depression [32]. Patients in the maternity ward selected one of the four answers in each statement that best described their well-being over the previous seven days. The results (100% response rate) were summed according to the following key: the response options for questions 1, 2, and 4 were assigned points from 0 to 3, while the response options for the remaining questions (3 and 5 to 10) were assigned a score in reverse order from 3 to 0. The total possible score ranges from 0 to 30 points. A score of ≥10 points is borderline and may suggest emotional problems in the mother. A score of ≥12–13 points indicates the likelihood of a postpartum depression and requires psychiatric consultation. Regardless of the total score, a positive answer to the final statement “*I sometimes thought about harming myself*”, always requires a psychiatric consultation [21,24]. Cronbach’s alpha for the present sample was 0.84.

### 2.3. SWLS

The Satisfaction with Life Scale by Diener et al. [33], adapted in Polish by Jankowski [34] (pl. Skala Satysfakcji z Życia autorstwa Dienera i wsp., w polskiej adaptacji Konrada Jankowskiego), consists of five statements. Respondents were asked to rate the extent to which they agreed with each statement on a scale from 1 to 7 points. The obtained score (100% response rate) were summed according to the following key: the response options for questions 1, 2, and 4 were assigned points from 0 to 3, while the response options for the remaining questions (3 and 5 to 10) were assigned a score in reverse order from 3 to 0. indicates an overall feeling of satisfaction with one’s life. The total score ranges from 5 to 35 points. A higher number of points indicates greater satisfaction with one’s life. The raw score is converted into standardized units as follows: 5–9 points (sten 1), 10–11 (sten 2), 12–14 (sten 3), 15–17 (sten 4), 18–20 (sten 5), 21–23 (sten 6), 24–26 (sten 7), 27–28 (sten 8), 29–30 (sten 9), 31–35 (sten 10). The interpretation of the sten scale scores is as follows: 1–4 sten (low scores), 5–6 sten (average scores), 7–10 sten (high scores) [35]. Cronbach’s alpha for the present sample was 0.83.

### 2.4. EQ-5D-5L

The Euro-Quality of Life Questionnaire version 5L (pl. Kwestionariusz dotyczący zdrowia EQ-5D w wersji 5L), consists of two parts. The first part includes five categories: mobility; self-care; usual activities (e.g., work, study, household activities, family activities and leisure activities); pain/discomfort; and anxiety/depression. Five statements are assigned to each category: no problems; minor problems/slight severity; moderate problems/moderate severity; serious problems/significant severity; inability to perform the activity/extreme severity. Respondents selected the statement that best described their health on the day the questionnaire was completed. To interpret the results, each of the five statements was assigned a level from 1 to 5. The results (100% response rate) are generally presenteded separately for each of the five levels; the resulting scores are combined into a single 5-digit number that describes the respondent’s health status. When analyzing the responses in this study, it was decided to combine levels 2 with 3 and levels 4 with 5. The second part of the questionnaire is the Visual Analogue Scale (EQ-VAS), which is used for the subjective assessment of one’s own health. Respondents marked their current health status on the scale, with 0 meant the worst imaginable health and 100 meant the best imaginable health. For this study, the 100-point scale was divided into four equal parts [36,37,38,39,40]. Cronbach’s alpha for the present sample was 0.83.

### 2.5. Statistical Analysis

The database was developed using Microsoft Excel 2013. The statistical analysis was performed using Statistica 13.3 (StatSoft, Cracow, Poland). Statistical significance was determined at *p* < 0.05 level. Quantitative data were presented using descriptive characteristics (mean ± standard deviation/median, quartile 1–quartile 3), while qualitative data were presented as a quantity and percentage. Correlations between qualitative characteristics were analyzed using the chi-squared test (χ^2^), and ordinal characteristics were analyzed using the gamma correlation coefficient significance test. For quantitative characteristics, Spearman’s R correlation coefficient was used for correlation analysis, and the Mann-Whitney U and Kruskal-Wallis tests were used to test for significance of differences within groups (asymmetry was demonstrated using the Shapiro-Wilk test). Analyses were performed in groups depending on the research period in relation to the occurrence of the COVID-19 pandemic (before March 2020 and after the announcement of the first case of coronavirus in Poland on 4 March 2020 [41]). The analyses were performer according to the following factors: age, education, number of children, type of profession, material status, marital status, participation in childbirth classes, duration of pregnancy and type of delivery. A logistic regression model was used to estimate the risk of anxiety symptoms (over 8 points on the HADS-A scale) or depressive symptoms (over 8 points on the HADS-D scale or over 10 points on the EPDS scale). Crude odds ratios (ORs) with confidence intervals (95% CI) were calculated. Multivariate models (forward and backward stepwise method) included predictors that had a significant impact on the dependent variable and whose correlation strength with other variables was not significant. A statistical significance level of *p* < 0.05 was used as the criterion for including a variable in the model. Both methods gave the same results. Ordinal logistic regression (sten scores of 1–4, 5–6, 7–10 on the SWLS scale) was used to model the life satisfaction scale. A summary is presented in the diagrammatic form.

## 3. Results

### 3.1. Characteristics of the Study Group

The age of the respondents ranged from 18 to 45 years (mean age 30.3 ± 5.6 years). The largest group were women with higher education (*n* = 148; 48.6%), followed by those with vocational/secondary education (*n* = 136; 44.7%) and those with primary/lower secondary education (*n* = 20, 6.6%). Most of the respondents were married (*n* = 228; 75%), 72 (23.7%) were in an informal relationship, and four were single mothers (1.3%). Just under half of the respondents had two children (*n* = 144; 47.4%), ⅓ had one child (*n* = 93; 30.6%), and 67 (22%) had three or more children. In term of profession, 64.1% of respondents (*n* = 195) had a non-medical profession, 17 (5.6%) had a medical profession, and 92 (30.3%) did not work or were studying. Most respondents (*n* = 193; 63.5%) had an income per person below 3000 PLN, which is the minimum gross salary in Poland in 2020–2600 PLN [42]; 55 respondents (18.1%) were unable to specify their income or refused to answer. Almost ¾ did not participate in childbirth classes (*n* = 218; 71.7%).

Almost half of the patients participating in the study gave birth before the planned delivery date (*n* = 139; 45.7%), while approximately 40% gave birth on the planned date (*n* = 121; 39.8%). More than half of the pregnancies (*n* = 175; 57.6%) were terminated by cesarean section, in 140 women (46.1%) were planned operations. Planned cesarean section was significantly more common among women aged over 35 years of age (*p* = 0.0002), women who were mothers of three or more children (*p* = 0.0001), women with higher education (*p* = 0.003) and women with a higher economic status (*p* = 0.03) (see Table A1). The type of delivery was not associated with participation in a childbirth classes (*p* = 0.09). However, emergency cesarean sections were significantly more likely to be performed after the planned delivery date (*p* = 0.0001).

### 3.2. Mental State—The Main Results

**Figure 1:**
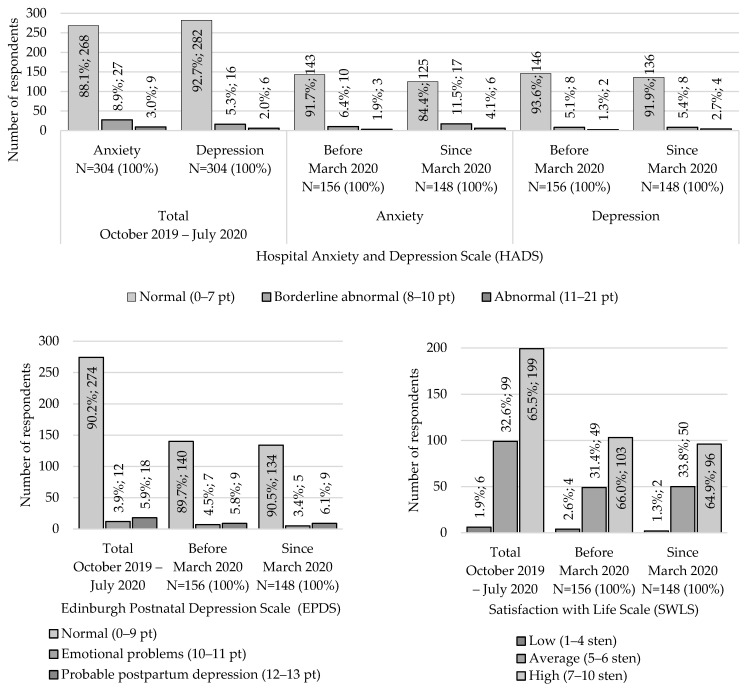
The emotional state of patients in the maternity ward at the Multispecialist District Hospital in Tarnowskie Góry throughout the study period (October 2019–July 2020) and in the periods before and after the announcement of the first case of coronavirus disease in Poland (March 2020).

The majority of respondents did not exhibit anxiety (*n* = 268; 88.1%, 95% CI: 84.0–91.6%) or depressive symptoms (*n* = 282; 92.8%, 95% CI: 89.3–95.4%) (see Figure 1). However, the proportion of subjects showing anxiety and depressive symptoms increased after the first coronavirus case was annoinced in Poland—the scores rose from 1.9% (95% CI: 0.4–5.5%) to 4.1% (95% CI: 1.5–8.6%) (*p* = 0.15) and from 1.3% (95% CI: 0.2–4.6%) to 2.7% (95% CI: 0.7–6.8%) (*p* = 0.66), respectively. The EDPS scores revealed 18 (5.9%, 95% CI: 3.6–9.2%) cases with symptoms of postpartum depression, including seven (2.3%, 95% CI: 0.9–4.7%) women who reported having had intrusive thoughts of self-harm. During the pandemic, the percentage of women with postpartum disorders increased from 5.8% (95% CI: 2.7–10.7%) to 6.1% (95% CI: 2.8–11.2%) (*p* = 0.88). Figure 1 shows that a high level of life satisfaction characterised 199 (65.5%, 95% CI: 59.8–70.8%) of the respondents, with a decrease in the proportion of those reporting high level of satisfaction from 66% (95% CI: 58.0–73.4%) to 64.9% (95% CI: 56.6–72.5%) (*p* = 0.69) in March 2020.

**Figure 2:**
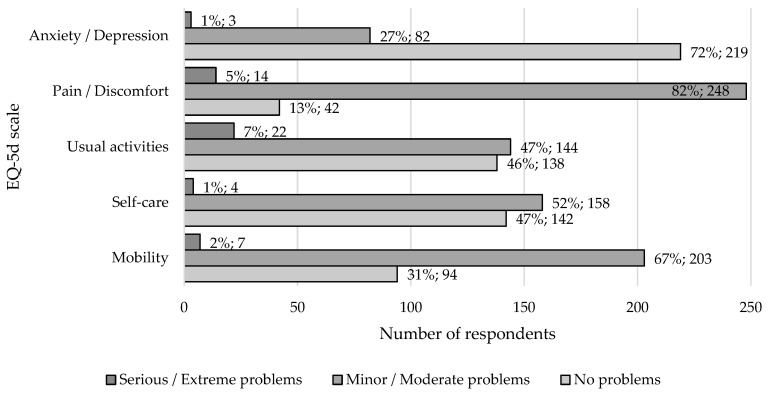
Quality of life dimensions according to the EQ-5D scale of patients in the maternity ward of the Multispecialist District Hospital in Tarnowskie Góry throughout the study period (October 2019–July 2020).

On the EQ-VAS scale from 0 to 100, the subjective assessment of the patients’ health status was at an average level of 76.0 ± 15.5 points. The majority of respondents (*n* = 192, 63.2%, 95% CI: 57.5–68.6%) raited their health status as ‘high’ (75–100 points), 1/3 as ‘good’ (50–75 points) (*n* = 98, 32.2%, 95% CI: 27.0–37.8%), and less than 5% rated is as ‘moderate’ (25–50 points) or ‘poor’ (0–25 points) (*n* = 11, 3.6%, 95% CI: 1.8–6.4%/*n* = 3, 1.0%, 95% CI: 0.2–2.9%, respectively). Analysis of quality of life aspects of postpartum women, according to the EQ-5D questionnaire, indicates an increased prevalence of pain problems (*n* = 262, 86.2%, 95% CI: 81.8–89.9%), mobility problems (*n* = 210, 69%, 95% CI: 63.6–74.2%) and performance of usual activities (*n* = 166, 55%, 95% CI: 48.8–60.3%). Serious problems with anxiety and depressive disorders occurred in three women (1%) (Figure 2).

### 3.3. Mental State—The Subgroup Results

Self-rated health status was significantly correlated with rates of mobility (R’ = −0.43, *p* < 0.0001), self-care (R’ = −0.42, *p* < 0.0001), performing usual activities (R’ 0.41, *p* < 0.0001), experiencing pain and discomfort (R’ = 0.44, *p* < 0.0001) and anxiety and depression (R’ = −0.33, *p* < 0.0001). Furthermore, significantly lower health status scores were reported by women with higher education (*p* = 0.0300), giving birth before the planned delivery date (*p* = 0.0200), and those who underwent an emergency cesarean section (*p* < 0.0001) (Table A2).

Women who had undergone a cesarean section, whether planned or emergency, were significantly more likely to report problems with mobility, self-care, and performing usual activities. They were also more likely to experiencing pain/discomfort and anxiety/depression. A significantly higher level of health was reported by women who gave birth naturally than those who had a cesarean section (Table 1). Additionally, women who had given birth for the first time were significantly more likely to experience problems with mobility, self-care, performing usual activities as well as experiencing severe pain. No significant variations in the quality of life dimensions was observed between age groups or occupational groups.

A moderate, significant correlation was confirmed between the EPDS questionnaire scores and HADS and EQ-5D scores (Table 2). Furthermore, a significant, negative correlation was observed between the level of life satisfaction and the HADS-A (G = −0.37, *p* = 0.0006) and HADS-D (G = −0.45, *p* = 0.0005) scores.

A significantly higher risk of anxiety symptoms was observed among women who had a planned cesarean section, experienced severe problems or inability to move, took care of themselves, or performed usual activities. They also experienced severe pain and had a lower self-rated of theit health status (see Table 3). Depressive symptoms were significantly associated with severe problems or an inability to move or perform usual activities, as well as a low self-rated health status. A significantly higher risk of postpartum depression was associated with having serious problems or inability to care for themselves, experiencing severe pain and having a low self-rated health status. In contrast, higher levels of life satisfaction were associated with higher levels of education and economic status, attendance at childbirth classes, and a better self-rated health status (see Table 3). The presence of anxiety and depressive symptoms significantly reduced life satisfaction.

**Figure 3:**
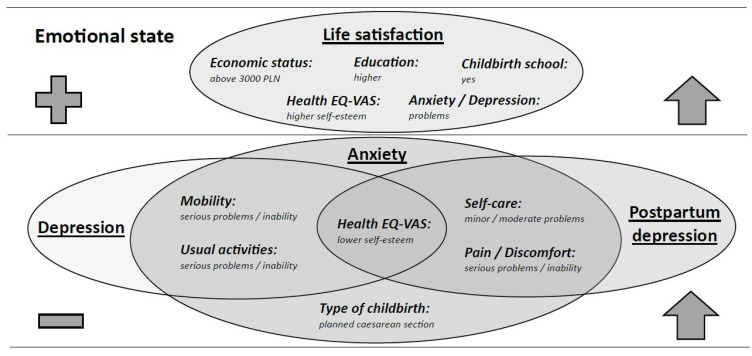
Venn Diagram: Factors important for the emotional state of breastfeeding women.

The factors relevant to the emotional state of breastfeeding women were identified (see Figure 3).

Applying a multivariate stepwise backward logistic regression model showed that the risk of anxiety symptoms (OR = 0.96, 95% CI: 0.94–0.98), depressive symptoms (OR = 0.96, 95% CI: 0.93–0.98) and postpartum depression symptoms (OR = 0.97, 95% CI: 0.95–0.99) decreased with increasing self-rated health. A higher level of life satisfaction was significantly associated with higher education (OR = 3.98, 95% CI: 1.23–12.85), higher economic status (OR = 3.12, 95% CI: 1.42–6.86), attending a childbirth classes (OR = 1.99, 95% CI: 1.02–3.89) and higher self-assessed health (OR = 1.04, 95% CI: 1.03–1.06).

**Figure 4:**
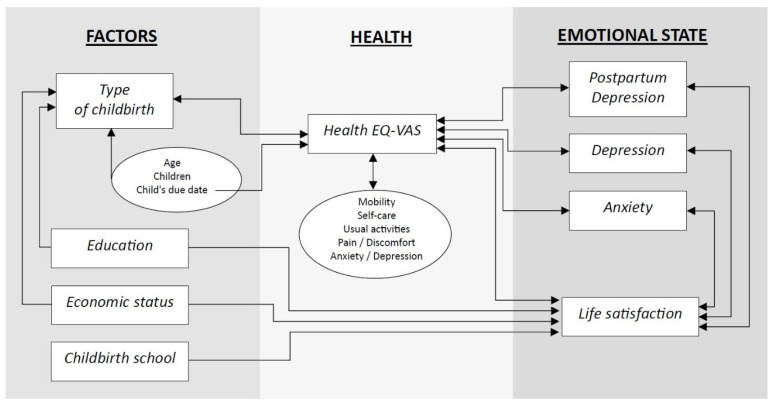
Relational diagram for the emotional state of breastfeeding women.

Finally, a relational diagram was constructed that showed the link between the emotional state of breastfeeding women and their health status, taking into account confounding factors (Figure 4).

## 4. Discussion

Many numerous physical, hormonal, emotional and psychological changes occur in the female body during the postpartum perion. The most significant changes occur in the first few days after delivery and are associated with individual psychological vulnerability. Although many risk factors for postpartum mental health disorders have been identified, but a large number of cases remain undiagnosed and untreated due to low awareness in society. Early identification of factors increasing the risk of mood disorders should begin during pregnancy [17,43]. Despite the organizational standard for perinatal care in Poland since 2019, which requires three assessments of women’s mental health during pregnancy and postpartum, there is still a lack of specific guidelines for screening, diagnosis, and treating postpartum depression [22]. The nationwide programme for the prevention of postpartum depression, implemented by the Ministry of Health, was only conducted by nine institutions in seven voivodeships [44]. This programme was not implemented in the Silesian Voivodeship, where this study was conducted. A 2015 literature review indicates that the worldwide prevalence of postpartum depressive episodes ranges from 0.1% in Finland to 26.3% in India [45]. A subsequent systematic review and meta-analysis showed that the highest incidence rates of postpartum depression were found in South Africa (40%), followed by South Asia (22.3%), South America (21.7%), West Asia (19.8%), and North Africa (18.4%) [46]. The incidence of postpartum depression has increased from 9.4% in 2010 to 19% in 2021 [47]. The increased risk of postpartum depression may persist for up to a year after delivery [43]. A Swedish study reported that 10% of women experienced postpartum depression within 1.5 years after delivery [48].

Patients in maternity wards with a result indicating an increased risk of mental disorders should have the opportunity to a consult with a hospital psychologist or psychiatrist so that appropriate treatment can be implemented as soon as possible. Analyses of numerous studies report that early intervention significantly reduces the proportion of women develop postpartum depression [49]. A study by Augustyniak et al. indicates that mood changes, tearfulness, and fatigue were significantly more common among those not receiving postpartum education [50]. Therefore, it is crucial to promote up-to-date knowledge of diagnostic and treatment methods treating emotional and mood disorders among medical personnel who have direct contact with women in the early postpartum period—such as midwives, obstetricians, neonatologists as well as among those closest to them [49,51]. Additionally, actions should be taken to facilitate access to free psychological and psychiatric counseling [52].

14.5% of postpartum women in the gynecology and obstetrics wards of the Poznań University of Medical Sciences Clinical Hospital, Poland scored higly on the Edinburgh Postpartum Depression Scale, indicating emotional distress. 8.9% were likely to have postpartum depression [53]. By comparison, in our study, emotional problems were reported by 3.9% of postpartum women, and probable postpartum depression by 5.9%. Similarly, thoughts of self-harm were reported by 3% of women in Poznań and 2.3% in our study. Regarding depressive disorders as assessed by the Hospital Anxiety and Depression Scale, 92.8% of the Poznań postpartum women had a normal scores, 4.9% had borderline symptoms, and 2.3% had a score indicating depressive disorder. In our study, 92.8% of respondents were within the normal range, borderline states were observed in 5.3%, and depressive disorders in 2%. no anxiety disorders were found in 79.1% of postpartum women from Poznań, mild anxiety states were observed in 13.5%, and anxiety disorders in 7.4%. In our study, 88.2% of respondents were in the normal range, 8.9% had mild anxiety states, and 3% had anxiety disorders. A study conducted at the Medical University of Warsaw, involving a group of 300 maternity ward patients, reported that 35% of women had a score indicating probable postpartum depression, compared to 5.9% in our study [54].

As in other studies, the present study found no evidence to suport the hypothesis that age is a risk factor for postpartum depression [53,54]. A literature review as well as the results of our study, there is no correlation between the number of children a woman has had, duration of her pregnancies and her results on the Edinburgh Postpartum Depression Scale [53,54,55,56]. However, the study by Iracka et al. indicates that women giving birth for the first time are more frequently experience symptoms of postpartum depression, however this result was not statistically significant [57]. Nevertheless, the significant impact of the type of delivery on the reduced quality of life and health, as revealed by the presented study, it is noteworthly, as it directly influences the manifestation of anxiety and depressive symptoms.

A cesarean section usually results in the woman being immobilized for a longer period than a vaginal delivery. This can lead to a lowered self-esteem and self-perception as a mother. A cesarean section, especially if performen as an emergency procedure, may be perceived by the mother as a failure or, conversely, as a lifesaving for the child’s health and life. Therefore, the attitude of medical staff toward women in laboring woman is crucial, as it can significantly impact the occurrence of postpartum emotional disorders. Scientific reports are inconsistent regarding the impact of the type of delivery on the emotional state of postpartum women. Own research and other Polish studies, do not confirm a statistically significant correlation between the mode of delivery and the occurrence of symptoms indicative of postpartum depression [53,54,55,56,58]. The literature suggests that the mode of delivery does not significantly affect higher scores on the Beck Depression Inventory [59]. A study by Adams et al., which used the Hopkins Symptom Checklist-25 (HSCL-25) questionnaire to assess anxiety and depression levels, also found no relationship between the type of delivery and the occurrence of anxiety and depressive disorders [60]. In turn, a Polish study by Czerwińska-Osipiak showed that emotional disturbances during the postpartum period were more prevalent among women who had a cesarean section compared to women who gave birth naturally [61]. It should also be noted that in our study, the risk of anxiety symptoms was significantly higher among women who had planned cesarean section. In the studies by Kowalska and Iracka, symptoms of postpartum depression were slightly more common among women who gave birth by cesarean section compared to women who gave birth naturally [57,62]. Blom et al. also indicate that an emergency cesarean section may increase the risk of postpartum depression [63]. Additionally, Grisbrook et al. report that an emergency cesarean section is associated with symptoms of post-traumatic stress disorder symptoms, which indirectly increases the risk of postpartum depression [64]. A 2017 meta-analysis of 28 studies suggests that cesarean delivery, particularly emergency delivery, may increase the risk of postpartum depression. The authors also emphasize the need for further research in this area [65]. However, a more recent literature review does not confirm an association between cesarean delivery and postpartum depression [66].

The presented study, as well as the study by Gebuza et al., did not confirm the effect of the type of delivery on perceived life satisfaction, measured by the SWLS scale [67]. However, the results of a study conducted in Lublin in 2020 indicated a high level of life satisfaction among 77.6% of women who gave birth naturally and 61.4% of women who had a cesarean section [68]. This study also showed that single mothers had lower SWLS scores, and a higher level of education and a better material situation were associated with higher life satisfaction scores. Our own study confirmed the influence of education and economic status, but also indicated a positive association with participation in childbirth classes and a better self-rated of health status. Furthermore, it was demonstrated that anxiety and depressive symptoms significantly reduced life satisfaction.

The main benefits of having a partner present at birth are primarily indicated psychological support and a deepening of the bond between partners [69]. Shortly after the official announcement of the COVID-19 pandemic in Poland, hospitals introduced restrictions prohibiting births with an attendant. Birth without the support of a loved one were often associated with higher level of stress and anxiety for the mother [70,71]. This relationship is confirmed by our study, revealing that anxiety and depression were more frequently observed during the pandemic.

Even before the outbreak of the global COVID-19 pandemic, depressive and anxiety disorders were among the leading causes of health issues worldwide. The pandemic itself and preventative measures introduced to slow the spread of the virus have impacted on the mental health of the general population, but the scientific evidence regarding the extend of this impact is inconsistent. Robinson et al. report that the impact was small and that the situation stabilized over time [72]. However, according to estimates by the ‘COVID-19 Mental Disorders Collaborants’, there was a 276% increase in severe depressive and anxiety disorders in 2020 compared to the pre-pandemic period. This was due to the rising number of SARS-CoV-2 infections and social isolation [73].

As study from Ireland at the beginning of the pandemic reports that, between February 2019 and March 2020, there was no increase in new cases of depression or anxiety disorders [74]. Gori and Topino showed that the level of perceived worry, state anxiety and stress remained unchanged between March 2020 and March 2021 [75].

While the examples cited refer to the general population, when the analysis is limited to the group of women studied, the consequences of lockdowns are significantly more serious. Scientific reports demonstrate that infectious disease epidemics can also negatively impact mental health, especially during the perinatal period [76]. Depressive and anxiety symptoms were significantly more severe during the pandemic [77,78]. Caffieri et al. point out that although the COVID-19 pandemic was probably not the only factor worsening mental health in the postpartum period, it is recommended that public health efforts worldwide should prioritize perinatal depression and anxiety [79].

The strength of the study is that a large study group was surveyed using several questionnaires in the context of mental health.

## 5. Limitations of the Study

Among the limitations, it is worth be noted that the hospital in which the study was conducted has II of III reference level. Reference levels are used to classify hospitals based on their ability to perform specific medical procedures. Thus, this hospital provides care, among women with high-risk pregnancies and births can take place from the 32nd week of pregnancy. Therefore, almost half of the study group gave birth before the planned date, and 57.6% of deliveries were by cesarean section. However, it is worth mentioning that, according to data from the National Health Fund, the nationwide cesarean section birth rate in 2023 was 48%, compared to 50% in the Silesian Voivodeship [80]. Another limitation may have been the health status of the participants, which, according to the results, had a significant impact on the occurrence of anxiety and depressive disorders. The questionnaire did not ask about the presence of chronic diseases. Therefore, it was not possible to determine whether the low self-assessment of health was due to the condition immediately after delivery or was related to an exacerbation of the illness. The timing of the survey was also relevant to the study. The onset of the COVID-19 pandemic significantly changed the functioning of hospital units. This was evidenced by the increase in anxiety and depressive disorders among postpartum women.

## 6. Conclusions

Most women in the maternity ward reported good psychological well-being. Anxiety symptoms affected 11.9% of women, depressive symptoms affected 7.3%, and postpartum depression symptoms affected 5.9%. The COVID-19 pandemic has led to an increase in anxiety and depressive behaviors.

Women who gave birth by cesarean section were more likely to declare problems that reduced the quality of their life and health than women who gave birth naturally.

The risk of experiencing of anxiety, depressive and postpartum depression symptoms decreased as self-rated health increased. Higher levels of life satisfaction were associated with higher levels of education and economic status, attendance at childbirth classes, and higher self-rated health.

Screening for mental disorders during the postpartum period enables the early identification of symptoms and the implementation of appropriate treatment. Particular attention should be paid to women who give birth by cesarean section and those with health compaints.

## Figures and Tables

**Table 1:** The frequency of problems reducing the quality of life, measured by the EQ-5D scale, in groups categorised by the type of delivery.

EQ-5D	Total	Type of Childbirth			*p*-Value	Children			*p*-Value
		Natural Childbirth	Planned Caesarean Section	Emergency Cesarean Section		1	2	3+	
		129 (100)	140 (100)	35 (100)		93 (100)	144 (100)	67 (100)	
Mobility	No problems	59 (45.7)	28 (20)	7 (20)	<0.0001	21 (22.6)	39 (27.1)	34 (50.8)	<0.0001
	Minor/Moderate	68 (52.7)	111 (79.3)	24 (68.6)		68 (73.1)	102 (70.8)	33 (49.3)	
	Serious/Extreme	2 (1.6)	1 (0.7)	4 (11.4)		4 (4.3)	3 (2.1)	0 (0)	
Self-care	No problems	82 (63.6)	49 (35)	11 (31.4)	<0.0001	36 (38.7)	63 (43.8)	43 (64.2)	<0.0001
	Minor/Moderate	47 (36.4)	90 (64.3)	21 (60)		54 (58.1)	80 (55.6)	24 (35.8)	
	Serious/Extreme	0 (0)	1 (0.7)	3 (8.6)		3 (3.2)	1 (0.7)	0 (0)	
Usual activities	No problems	77 (59.7)	50 (35.7)	11 (31.4)	0.0005	39 (41.9)	60 (41.7)	39 (58.2)	0.0030
	Minor/Moderate	47 (36.4)	77 (55)	20 (57.1)		43 (46.2)	77 (53.5)	24 (35.8)	
	Serious/Extreme	5 (3.9)	13 (9.3)	4 (11.4)		11 (11.8)	7 (4.9)	4 (6)	
Pain/ Discomfort	No problems	26 (20.2)	14 (10)	2 (5.7)	0.0200	14 (15.1)	14 (9.7)	14 (20.9)	0.0400
	Minor/Moderate	99 (76.7)	120 (85.7)	29 (82.9)		72 (77.4)	124 (86.1)	52 (77.6)	
	Serious/Extreme	4 (3.1)	6 (4.3)	4 (11.4)		7 (7.5)	6 (4.2)	1 (1.5)	
Anxiety/ Depression	No problems	103 (79.8)	94 (67.1)	22 (62.9)	0.0400	71 (76.3)	96 (66.7)	52 (77.6)	0.9000
	Minor/Moderate	26 (20.2)	44 (31.4)	12 (34.3)		21 (22.6)	46 (31.9)	15 (22.4)	
	Serious/Extreme	0 (0)	2 (1.4)	1 (2.9)		1 (1.1)	2 (1.4)	0 (0)	
Health EQ-VAS	[pkt]	80 (75–90)	75 (65–85)	70 (55–80)	<0.0001	80 (65–90)	79.5 (70–85)	80 (70–90)	0.1300

Data presented as numbers and percentages *n* (%); *p*-value according to the X^2^ test; EQ-5D—Euro-Quality of Life Questionnaire.

**Table 2:** Compatibility of the results of questionnaires assessing the emotional state of patients in the maternity ward of the Multispecialist District Hospital in Tarnowskie Góry.

Scale		Total	Edinburgh Postnatal Depression Scale (EPDS)			Gamma Coefficient	*p*-Value
			Normal	Emotional Problems	Probable Postpartum Depression		
		304 (100)	274 (90.1)	12 (3.9)	18 (5.9)		
HADS-A	Normal	268 (88.2)	256 (84.2)	7 (2.3)	5 (1.6)	0.9	<0.0001
	Borderline abnormal	27 (8.9)	17 (5.6)	4 (1.3)	6 (2)		
	Abnormal	9 (3)	1 (0.3)	1 (0.3)	7 (2.3)		
HADS-D	Normal	282 (92.8)	264 (86.8)	9 (3)	9 (3)	0.88	<0.0001
	Borderline abnormal	16 (5.3)	9 (3)	2 (0.7)	5 (1.6)		
	Abnormal	6 (2)	1 (0.3)	1 (0.3)	4 (1.3)		
EQ-5D Anxiety/Depression	No problems	219 (72)	214 (70.4)	3 (1)	2 (0.7)	0.89	<0.0001
	Minor/Moderate problems	82 (27)	60 (19.7)	8 (2.6)	14 (4.6)		
	Serious/Extreme problems	3 (1)	0 (0)	1 (0.3)	2 (0.7)		
SWLS	Low	6 (2)	4 (1.3)	1 (0.3)	1 (0.3)	−0.56	<0.0001
	Average	99 (32.6)	82 (27)	7 (2.3)	10 (3.3)		
	High	199 (65.5)	188 (61.8)	4 (1.3)	7 (2.3)		

Data presented as numbers and percentages *n* (%); *p*-value according to the Gamma coefficient test; EPDS—Edinburgh Postnatal Depression Scale; HADS-A, HADS-D—Hospital Anxiety and Depression Scale (HADS); EQ-5D—Euro-Quality of Life Questionnaire; SWLS—Satisfaction with Life Scale.

**Table 3:** Crude odds ratios (OR) with 95% confidence interval (CI) for the relationship between the occurrence of anxiety disorders (8 points or more on the HADS-A), depression (8 points or more on the HADS-D), and postpartum depression (10 points or more on the EPDS), and life satisfaction (on the SWLS).

Data	Reference Group	Level	Anxiety HADS-A		Depression HADS-D		Postpartum Depression EPDS		Life Satisfaction SWLS	
			OR (95% CI)	*p*-Value	OR (95% CI)	*p*-Value	OR (95% CI)	*p*-Value	OR (95% CI)	*p*-Value
Age	under 25	25–35	0.86 (0.33–2.24)	0.7500	1.18 (0.33–4.23)	0.8000	0.77 (0.27–2.18)	0.6200	1.52 (0.8–2.92)	0.2000
		35+	0.83 (0.25–2.78)	0.7600	0.84 (0.16–4.38)	0.8400	1.22 (0.36–4.14)	0.7500	2.04 (0.89–4.71)	0.0900
Education	primary/secondary school	vocational/ secondary	1.84 (0.23–14.96)	0.5700	0.64 (0.13–3.19)	0.5800	2.18 (0.27–17.55)	0.4600	1.97 (0.78–4.99)	0.1500
		higher	3.5 (0.45–27.42)	0.2300	0.72 (0.15–3.53)	0.6900	2.14 (0.27–17.16)	0.4700	4.1 (1.6–10.51)	0.0030
Marital status	single mother	informal relationship	0.38 (0.04–4.05)	0.4200	0.22 (0.02–2.57)	0.2300	0.54 (0.05–5.69)	0.6100	1 (0.14–7.29)	1
		married	0.4 (0.04–4.01)	0.4400	0.23 (0.02–2.3)	0.2100	0.26 (0.03–2.6)	0.2500	2.18 (0.31–15.37)	0.4400
Children	1	2	1.34 (0.52–3.47)	0.5400	2.73 (0.75–9.94)	0.1300	1.65 (0.65–4.13)	0.2900	0.65 (0.37–1.13)	0.1200
		3+	1.22 (0.39–3.82)	0.7300	3.5 (0.87–14.07)	0.0800	1.21 (0.39–3.77)	0.7500	0.8 (0.41–1.57)	0.5200
Economic status	under 3000 PLN	above 3000 PLN	0.55 (0.16–1.95)	0.3600	1.53 (0.56–4.2)	0.4000	0.29 (0.07–1.26)	0.1000	2.96 (1.41–6.24)	0.0040
Profession	non-medical	medical	2.15 (0.56–8.28)	0.2600	0.69 (0.09–5.56)	0.7300	0.57 (0.07–4.56)	0.6000	1.09 (0.37–3.23)	0.8800
Childbirth school	no	yes	1.31 (0.62–2.76)	0.4800	0.73 (0.26–2.04)	0.5500	1.1 (0.48–2.5)	0.8300	1.99 (1.13–3.49)	0.0200
Child’s due date	before the planned date	on the planned date	1.01 (0.43–2.35)	0.9800	1.16 (0.47–2.89)	0.7500	1.08 (0.48–2.39)	0.8600	1.02 (0.61–1.7)	0.9500
		after the planned date	0.72 (0.19–2.64)	0.6200	0.61 (0.13–2.92)	0.5400	0.65 (0.18–2.39)	0.5200	0.85 (0.43–1.72)	0.6600
Type of childbirth	natural childbirth	planned caesarean section	3.75 (1.35–10.44)	0.0100	2.1 (0.77–5.7)	0.1500	1.65 (0.72–3.74)	0.2400	1.28 (0.77–2.11)	0.3400
		emergency cesarean section	3.37 (0.85–13.32)	0.0800	1.92 (0.46–8.11)	0.3700	1.12 (0.29–4.3)	0.8700	0.91 (0.42–1.95)	0.8100
EQ-5D Mobility	no problems	minor/moderate problems	1.15 (0.46–2.87)	0.7700	1.42 (0.5–4.03)	0.5100	0.98 (0.42–2.25)	0.9500	0.9 (0.54–1.51)	0.6900
		serious problems/ inability	12.14 (2.06–71.73)	0.0100	7.12 (1.1–46.24)	0.0400	3.78 (0.64–22.36)	0.1400	1.26 (0.23–6.89)	0.7900
EQ-5D Self-care	no problems	minor/moderate problems	2.09 (0.87–5.02)	0.1000	2.02 (0.8–5.11)	0.1400	2.71 (1.17–6.3)	0.0200	0.82 (0.51–1.31)	0.4000
		serious problems/ inability	16.5 (2.05–132.85)	0.0100	-	-	-	-	1.45 (0.15–14.43)	0.7500
EQ-5D Usual activities	no problems	minor/moderate problems	1.66 (0.67–4.15)	0.2800	1.4 (0.52–3.78)	0.5100	1.71 (0.76–3.89)	0.2000	0.87 (0.54–1.42)	0.5800
		serious problems/ inability	6.4 (1.96–20.95)	0.0020	5.5 (1.57–19.29)	0.0080	2.02 (0.51–8.01)	0.3200	1.77 (0.61–5.1)	0.2900
EQ-5D Pain/Discomfort	no problems	minor/moderate problems	2.01 (0.46–8.88)	0.3600	1.47 (0.33–6.62)	0.6100	1.95 (0.44–8.61)	0.3800	1.2 (0.61–2.36)	0.6000
		serious problems/ inability	6.67 (0.97–45.92)	0.0500	5.46 (0.81–36.82)	0.0800	15 (2.55–88.17)	0.0030	0.37 (0.11–1.25)	0.1100
EQ-5D Anxiety/Depression	no problems	minor/moderate problems	-	-	-	-	-	-	0.5 (0.3–0.84)	0.0090
		serious problems/ inability	-	-	-	-	-	-	0.02 (0.002–0.24)	0.0020
Health EQ-VAS			0.96 (0.94–0.99)	0.0010	0.96 (0.94–0.99)	0.0010	0.96 (0.93–0.98)	0.0004	1.01 (1.01–1.04)	0.0010

Results presented as crude odds ratios (OR) with 95% confidence interval (CI); *p*-value according to the Wald test; EPDS—Edinburgh Postnatal Depression Scale; HADS-A, HADS-D—Hospital Anxiety and Depression Scale (HADS); EQ-5D—Euro-Quality of Life Questionnaire; SWLS—Satisfaction with Life Scale.

## Data Availability

The data presented in this study are available on request from the corresponding author. The data are not publicly available due to privacy reasons.

## Abbreviations

The following abbreviations are used in this manuscript:

BDI	Beck Depression Inventory
CI	Confidence intervals
COVID-19	Coronavirus disease 2019
DSM-5	Diagnostic and Statistical Manual of Mental Disorders
EPDS	Edinburgh Postnatal Depression Scale
EQ-5D	Euro-Quality of Life Questionnaire
EQ-VAS	Visual Analogue Scale
HADS	Hospital Anxiety Depression Scale
HRQoL	Health-related quality of life
HSCL-25	Hopkins Symptom Checklist-25
ICD-10/ICD-11	International Statistical Classification of Diseases and Related Health Problems
MAMA	Maternal Adjustment and Maternal Attitudes
MGI	Mother Generated Index
OR	Crude odds ratio
PDSS	Postpartum Depression Screening Scale
PNMI	Postnatal Morbidity Index
PPD	Postpartum depression
SF-36	36-Item Short-Form Health Survey
SRRS	Social Readjustment Rating Scale
SWLS	Satisfaction with Life Scale

## Appendix A

**Table A1:** Types of childbirth grouped by age, socio-economic factors and child’s due date.

Data		Type of Childbirth			*p*-Value
		Natural Childbirth	Planned Caesarean Section	Emergency Caesarean Section	
Age	under 25	21 (46.7)	12 (26.7)	12 (26.7)	0.0002
	25–35	94 (45.6)	92 (44.7)	20 (9.7)	
	35+	14 (26.4)	**36 (67.9)**	3 (5.7)	
Education	primary/secondary school	16 (80)	3 (15)	1 (5)	0.0030
	vocational/secondary	60 (44.1)	58 (42.7)	18 (13.2)	
	higher	53 (35.8)	**79 (53.4)**	16 (10.8)	
Marital status	single mother	2 (50)	0 (0)	2 (50)	0.0500
	informal relationship	33 (45.8)	28 (38.9)	11 (15.3)	
	married	94 (41.2)	112 (49.1)	22 (9.7)	
Children	1	39 (41.9)	28 (30.1)	26 (28)	0.0001
	2	62 (43.1)	75 (52.1)	7 (4.9)	
	3+	28 (41.8)	**37 (55.2)**	2 (3)	
Economic status	under 3000 PLN	86 (44.6)	86 (44.6)	21 (10.9)	0.0300
	above 3000 PLN	14 (25)	**33 (58.9)**	9 (16.1)	
Living conditions	standard	8 (53.3)	6 (40)	1 (6.7)	0.2000
	good	54 (40)	59 (43.7)	22 (16.3)	
	very high	67 (43.5)	75 (48.7)	12 (7.8)	
Job	learning/studying	8 (53.3)	3 (20)	4 (26.7)	0.0200
	do not work	38 (49.4)	30 (39)	9 (11.7)	
	physical	35 (43.2)	33 (40.7)	13 (16.1)	
	mental	48 (36.6)	**74 (56.5)**	9 (6.9)	
Profession	non-medical profession	75 (38.9)	100 (51.8)	18 (9.3)	0.5300
	medical professsion	7 (41.2)	7 (41.2)	3 (17.7)	
Childbirth school	no	89 (40.8)	108 (49.5)	21 (9.6)	0.0900
	yes	40 (46.5)	32 (37.2)	14 (16.3)	
Child’s due date	before the planned date	45 (32.4)	**73 (52.5)**	**21 (15.1)**	0.0001
	on the planned date	50 (41.3)	**65 (53.7)**	6 (5)	
	after the planned date	34 (77.3)	2 (4.6)	**8 (18.2)**	

Data presented as numbers and percentages *n* (%); *p*-value according to the X^2^ test.

**Table A2:** Average self-assessment level of health status [points] of breastfeeding women grouped by age, socio-economic factors, child’s due date and type of delivery.

Data		EQ-VAS		*p*-Value
		X ± SD	Med. (Q1–Q3)	
Age	25–35	76.1 ± 15.1	80 (70–90)	0.2400
	under 25	78.4 ± 16.3	80 (70–90)	
	35+	73.4 ± 16.5	75 (60–85)	
Education	primary/secondary school	79.5 ± 13.9	80 (72.5–92.5)	0.0300
	vocational/secondary	78.1 ± 14.7	80 (70–90)	
	higher	73.5 ± 16.2	80 (65–85)	
Marital status	informal relationship	77.1 ± 15.1	80 (70–90)	0.2600
	married	75.9 ± 15.5	80 (70–90)	
	single mother	61.3 ± 22.9	52.5 (47.5–75)	
Children	2	74.7 ± 15.1	79.5 (70–85)	0.1400
	1	75.7 ± 17.6	80 (65–90)	
	3+	79.2 ± 13	80 (70–90)	
Economic status	under 3000 PLN	76.2 ± 15	80 (70–90)	0.8000
	above 3000 PLN	76.7 ± 14.9	80 (70–90)	
Living conditions	standard	76.3 ± 14.5	80 (70–90)	0.0600
	good	74.1 ± 15	75 (65–85)	
	very high	77.6 ± 16	80 (70–90)	
Job	physical	76.4 ± 16.7	80 (65–90)	0.3500
	do not work	78.2 ± 13.6	80 (70–90)	
	mental	74.3 ± 15.7	80 (70–85)	
	learning/studying	76.9 ± 16.9	80 (70–90)	
Profession	non-medical profession	75 ± 16.4	80 (70–90)	0.8600
	medical profession	75.4 ± 13	80 (65–85)	
Childbirth school	no	76 ± 15.5	80 (70–90)	0.9500
	yes	75.9 ± 15.8	80 (70–90)	
Child’s due date	before the planned date	73.7 ± 16.2	76 (65–85)	0.0200
	on the planned date	77 ± 14.4	80 (70–90)	
	after the planned date	80.4 ± 15.7	82.5 (72.5–90)	
Type of childbirth	natural childbirth	81.7 ± 12.2	80 (75–90)	<0.0001
	planned caesarean section	72.7 ± 16.4	75 (65–85)	
	emergency caesarean section	67.7 ± 16	70 (55–80)	

Data presented as: mean and standard deviation X ± SD, median and quartiles Med. (Q1–Q3); *p*-value according to the U Mann-Whitney test for 2 groups, the Kruskal-Wallis test for 3 or more groups.
